# Management Outcome and Factors Associated with Pediatric Surgical Patient Admitted to Arbaminch General Hospital, Southern Ethiopia, 2021: Retrospective Cross-Sectional Study

**DOI:** 10.1155/2022/6865874

**Published:** 2022-08-25

**Authors:** Fikre Moga, Filagot Assefa, Kalkidan Wondwossen, Yeshiwork Berhan

**Affiliations:** ^1^College of Medicine and Health Sciences, Arba Minch University, P.O. Box 21, Arba Minch, Ethiopia; ^2^Arba Minch College of Health Sciences, P.O. Box 155, Arba Minch, Ethiopia; ^3^College of Health Sciences, Arba Minch University, P.O. Box 100686, Addis Ababa, Ethiopia

## Abstract

**Background:**

Pediatric surgical situations are often disregarded around the world, particularly in developing countries. The number of children hospitalized for surgical reasons has climbed dramatically. There is extensive research on the management outcome of pediatric surgical admissions in industrialized countries, but developing countries have paid little attention to it. Furthermore, to the best of the authors' knowledge, there has been no research in the study area on the management outcome of pediatric surgical patient admission.

**Objective:**

To assess management outcome and factors associated with pediatric surgical patients admitted to Arbaminch General Hospital, Southern Ethiopia, 2021.

**Method:**

An institution-based retrospective cross-sectional study design was employed among 265 children with surgical problems. Data were collected from patients' medical records using pretested data collection checklist. Epi Data 4.2 was used to enter data, and data were exported to SPSS version 25 for analysis. Those variables with *P*-value ≤0.25 in bivariable analysis were entered into multivariable logistic regression analysis, and statistical significance was declared at *P* < 0.05.

**Result:**

A total of 265 surgically admitted children were included in this study. About 26% of study subjects were discharged with unfavorable management outcome. Children admitted due to trauma cause (AOR: 5.753, 95% CI: 2.366–13.987), children with a preexisting medical condition (AOR: 3.240, 95% CI: 1.436–7.310), children with an early complication (AOR: 2.515, 95% CI: 1.130–5.599), presenting to hospital ≥24 hr after the onset (AOR:8.351, 95% CI: 2.089–33.381), hospital stay >7 days (AOR: 10.671, 95% CI: 1.363–83.546), and children treated with surgery (AOR: 2.742, 95% CI: 1.137–6.611) were associated with unfavorable management outcome. *Conclusion and recommendations*: Twenty-six percent of patients were discharged with unfavorable outcome. Reasons for admission, preexisting medical condition, early complications, duration of hospital presentation, length of hospitalization, and type of management were all linked to the outcome of pediatric surgical admission. To have a good outcome, early identification and treatment of the cause are required, as well as well-equipped surgical care centers.

## 1. Introduction

Any clinical defect in children that necessitates invasive operations, excision, and wound closure is classified as a pediatric surgical disorder [1]. Surgically treatable diseases account for roughly 28–30% of the worldwide illness burden [2]. Around 67% of children and adolescents who require medical assistance around the world do not have timely access to surgical care [3]. In addition, death from postoperative complications was on the rise among children and adolescents [4]. Congenital malformations, injuries, cancer-related illnesses, gastrointestinal conditions, particularly appendicitis, and intussusceptions were the most common reasons for pediatric surgical admissions [5].

Surgical disorders that necessitate surgical and conservative therapy contribute significantly to the worldwide burden of mortality and morbidity rates, and this burden is exacerbated in economically impoverished countries. Even though children make up nearly half of a developing country's population, there is a slight focus on childhood surgical disorders, with attention instead being given to communicable disease and obstetrics care [6–8].

As a result, limited access to surgical care was linked to a high proportion of patient fatalities from easily curable surgical disorders. Eastern, western, and central Africa, as well as sub-Saharan Africa and South Asia, had a high unmet need for pediatric surgical care [2]. The lack of social support, lack of information on surgical conditions, conventional beliefs about disease processes, poor communication, and decreased quality of care were the key obstacles to improving pediatric surgical care in developing nations [9].

Approximately five billion people in the world lack timely access to surgical care. Every year, over 16.9 million individuals die as a result of surgical complications [2]. Around 1 million individuals died as a result of surgical complications, which is higher than malaria and AIDS combined [10].

According to the World Health Organization, almost 1.7 billion children did not have access to life-saving surgical care in 2017 [3]. Children's mortality, complication, and serious adverse event rates were 0.02 percent, 13.9 percent, and 5.7 percent, respectively, due to the surgical condition. Children's death rates were 5 to 15 times greater in poor countries than in industrialized countries [11]. In-hospital mortality is ten times higher in low- and middle-income nations than in high-income countries, which is linked to a lack of access to high-quality care [12]. In economically impoverished countries, less than 8% of children have access to surgical care [3]. In Africa, child mortality from pediatric surgical procedures is still significant, especially from congenital disorders (17%). In sub-Saharan African countries, it accounts for 6–12 percent of all pediatric admissions and 20% of pediatric outpatient visits [2].

The burden of most noncommunicable diseases in Ethiopia, including surgically curable ailments, has been steadily growing over time [13]. In some parts of Ethiopia, emergency admission is the most common way of pediatric admission. Children admitted on an elective basis (53.3%) were more likely to die than those admitted on an emergency basis (46.7%). Delay in presentation was found to be the most important factor in determining the outcome. More research on surgical problems is needed to determine the size of the negative outcome and the factors that contribute to it [14]. It is difficult to estimate the influence of surgical conditions on children's health in sub-Saharan African nations, especially Ethiopia, due to a lack of surgical care data [15].

In industrialized countries, there is ongoing study and data on the management outcome of pediatric surgical admissions and associated determinants, but poor countries, such as Ethiopia, have paid little attention to it. There is an insufficient study in Ethiopia, and to the best of the authors' knowledge, no investigation in the study area on the management outcome of pediatric surgical patient admission and its associated factors. Therefore, the aim of this study was to assess management outcome and factors associated with pediatric surgical patients admitted to Arbaminch General Hospital, Southern Ethiopia. The findings of this study are important in providing baseline data for hospitals and health bureaus to establish appropriate policies and strategies for enhancing pediatric surgical treatment. It is also significant for managers and policymakers to establish a plan of action for the pediatric surgical condition, and it can be used as a reference for nurse educators and researchers interested in conducting a future study in this area.

## 2. Methods and Materials

The study was carried out in Arbaminch General Hospital, which is found in Arbaminch town, Southern Ethiopia. The town is located 505 km away from Addis Ababa and 280 km from Awassa, a center of the southern nation nationality and people regional state (SNNPR). There is 1 zonal hospital, two health centers, and 69 private health institutions in Arba Minch town. The hospital's annual average total surgical admissions were 1220, with roughly 120 of them being children under the age of 18. The study was conducted in Arbaminch General Hospital from February 25 to March 25, 2021.

### 2.1. Study Design

An institution-based retrospective cross-sectional study design was employed.

### 2.2. Source Population

The source population for this study was all children less than 18 years who were admitted with a surgical problem and treated at Arbaminch General Hospital from January 1, 2017, to December 31, 2020.

### 2.3. Study Population

The study population for this study was all selected children less than 18 years old who were admitted for surgical care to Arbaminch General Hospital from January 1, 2017, to December 31, 2020, and those fulfilling inclusion criteria.

### 2.4. Inclusion Criteria

Children under the age of 18 who were admitted for surgical care and had a complete medical record were included in the study.

### 2.5. Exclusion Criteria

The study excluded children who had minor outpatient pediatric surgical procedures and those who were admitted for surgical care but departed against medical advice and were sent to another healthcare center for further therapy.

### 2.6. Sample Size Determination

Sample size of this study was calculated by using a single population proportion formula by taking a proportion of 80.5% favorable management outcome from the study conducted in Adama Medical Hospital [16], 95% confidence interval, and 5% significance level and by adding 10% for incomplete cards. Finally, the required sample size was found to be 265.

### 2.7. Sampling Technique

Four hundred sixty (460) pediatric patients were admitted to the surgical unit of Arbaminch General Hospital during the course of three years (from January 1, 2017, to December 31, 2020). There were 156 patients admitted in year one (from January 1, 2017, to December 31, 2018), 144 patients admitted in year two (from January 1, 2018, to December 31, 2019), and 160 patients admitted in year three (from January 1, 2019, to December 31, 2020). Then, based on the number of admitted patients, a specified sample was proportionally allotted to each year. Sampling frame was created based on a medical record in the hospital's patient registration book, and then a patient's medical card was randomly selected from the sampling frame using a systematic random sampling method, with every second medical chart serving as the sampling interval.

### 2.8. Operational Definitions

#### 2.8.1. Children

Consider the age group of less than 18 years.

#### 2.8.2. Favorable Outcome

Those admitted children with a surgical problem were discharged with improvement without any complications at the end of treatment.

#### 2.8.3. Unfavorable Outcome

Those surgically admitted children were discharged with different complications such as disfigurement and amputation and death at the end of treatment as recorded on the patients' cards.

### 2.9. Data Collection Instruments and Procedures

Data were collected from a patient's medical record by using pretested data collection checklist, which was prepared after reviewing different literature. Data were gathered from records of pediatric patient registration for the last three years (from January 1, 2017, to December 31, 2020). The checklist included sociodemographic characteristics, clinical data, time-related data, therapeutic related data, and the outcome of pediatric admission.

### 2.10. Data Quality Control

To ensure quality of the data, the checklist was pretested before one week of the main survey on 5% of the total sample. The collected data were revised and checked for completeness before data entry; then, incomplete data were discarded accordingly. One-day training was given to data collectors and the supervisor on the purpose of the study and how to collect data from patient card. During data collection period, adequate supervision was undertaken by supervisor and by principal investigator. Spot-checking for the collected checklist was done on a daily basis. The collected data were checked out for completeness, accuracy, and clarity by the principal investigator and supervisor.

### 2.11. Data Entry and Analysis

Data were entered using Epi Data 4.2 version and analyzed by using SPSS version 25. Descriptive statistics were performed for independent variables and the outcome variable. Logistic regression was performed, and all study variables with a *P*-value of ≤0.25 were entered into multivariable logistic regression. Adjusted odds ratio (AOR) with its respective 95% confidence interval (CI) was used, and statistical significance was declared at *P*-value of less than 0.05. Finally, findings of the results were presented using charts, graphs, and tables.

## 3. Results

### 3.1. Sociodemographic Characteristics

A total of 265 surgically admitted children were included in this study who were admitted between 2017 and 2020 GC. With a male to female ratio of 1.4 : 1, 157 (59.2%) study subjects were male (M157 : F108). The average age of the participants in the study was 7.37–5.776 (with a range of 1 to 17 years) (Table 1).

### 3.2. Cause and Forms of Pediatric Surgical Patient's Admission

**Figure 1:**
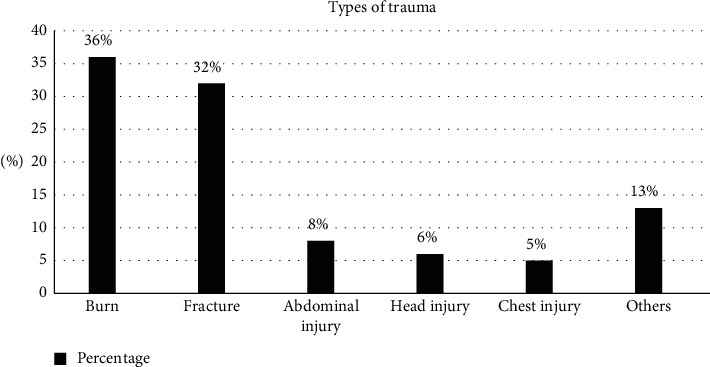
Type of trauma among pediatric surgical patients admitted to Arbaminch General Hospital from January 1, 2017, to December 31, 2020 (*n* = 265).

Trauma was the most common reason for admission among children, accounting for 99% of all admissions (Figure 1).

Surgical infection is the second most common reason for pediatric surgical admission, accounting for about 71% of all cases. Appendicitis, peritonitis, and osteomyelitis were the most often identified surgical infections, accounting for 39.4%, 18.3%, and 15.5% of those admitted due to surgical infection, respectively. The third most common reason for pediatric surgical admission was gastrointestinal problems, which accounted for around 48% of all cases. Intussusceptions and obstructions were the most common gastrointestinal conditions in children admitted to the hospital, accounting for 23 (47.9%) and 11 (22.9%) of all admitted children, respectively. Approximately 84% of traumatic causes are unintentional (Table 2).

### 3.3. Preexisting Medical Condition, Nutritional Status, and Clinical Presentation at the Time of Admission

Out of the total, 25.3% of the children had preexisting illnesses at the time of admission. The most often reported preexisting medical condition was anemia (19.3%), followed by epilepsy (13.9%), pneumonia (13.9%), asthma (9.5%), HIV/AIDS (8.0%), and diabetes (5%). About 61.0% of the children were malnourished when they were admitted. The majority of patients (60%) who were admitted to the hospital had a complaint of pain, followed by airway obstruction (67%), vomiting (64%), shock (bleeding) (62%), arrhythmia (42%), and loss of consciousness (38%).

### 3.4. Early Complication among Admitted Children

At the time of hospitalization, 115 (43.4%) of the children had already experienced an early complication. Among those who encountered early complications, local infection makes up 33 (12.5%), sepsis 22 (8.3%), shock 21 (7.9%), wound dehiscence 17 (6.4%), hematoma 16 (6.0%), and other conditions such pyelonephritis 6 (2.3%). Most patients who were admitted with an infection had unfavorable management outcome after being discharged.

### 3.5. Time-Related Factors of Pediatric Surgical Patient's Admission

Approximately 16.2% of hospitalized children visited the hospital early (within 24 hours) after the onset of the problem for hospital treatment, with a mean hospital stay of 23.11 days (SD: 20.826 with minimum and maximum of 1–119 days). About 32.5% of patients who stayed in the hospital for more than 7 days were discharged with unfavorable management outcome.

### 3.6. Therapeutic Intervention-Related Factors of Pediatric Surgical Admission

About 65.7% of hospitalized patients received surgical treatments, and 124 (71.3%) received major surgery. About 121 (69.5%) of all surgical procedures were carried out in emergency conditions. On patients who received conservative care, 90 (34%), 85 (32.1%), 63 (23.8%), 38 (14.3%), 25 (9.4%), and 19 (7.2%) used antibiotics, analgesics, wound washing and dressing, IV fluid replacement, orthopedic application, and tetanus antitoxoid, respectively.

### 3.7. Management Outcome of Pediatric Surgical Patient's Admission

**Figure 2:**
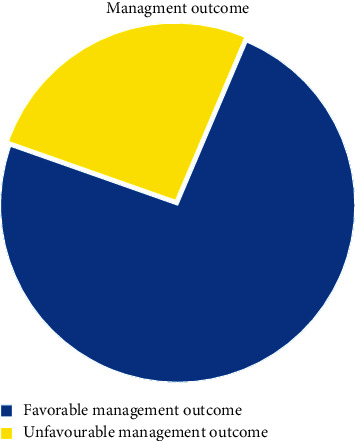
Management outcome of pediatric surgical patient's admission to Arbaminch General Hospital, Southern Ethiopia, 2021.

Twenty-nine (10.9%) of patients were discharged with significant comorbidity (body contracture, tracheostomy, and HTN), 13 (4.9%) had their limbs amputated, 8 (3.3%) had significant disfigurement, and 20 died, giving an overall mortality rate of 7.5% (Figure 2).

### 3.8. Factors Associated with Outcomes of Pediatric Surgical Patient's Admission

In bivariable logistic regression, age, sex, the residence of the patient, a form of admission, admission diagnosis, preexisting medical condition, nutritional status, early complication, time of hospital visit after the onset, length of hospital stay, and type of management were identified as associated factors with the management outcome of pediatric surgical patients.

After adjusting for potential confounders in multivariable logistic analysis, children admitted for trauma were 5.7 times more likely than those admitted for nontrauma reasons to be discharged with an unfavorable treatment outcome (AOR: 5.753, 95% CI: 2.366–13.987). Children who had a preexisting medical problem at the time of admission were 3.2 times more likely than their counterparts to be discharged with an unfavorable management outcome (AOR: 3.240, 95% CI: 1.436–7.310), and those who had an early complication at the time of admission were 2.5 times more likely to be discharged with an unfavorable management outcome than those who had no complication at the time of admission (AOR: 2.515, 95% CI: 1.130–5.599).

Children who arrived at the hospital after 24 hours of the onset were 8.3 times more likely to be discharged with unfavorable management outcome than children who arrived sooner (AOR: 8.351, 95% 2.089–33.381, children who stayed in the hospital for more than 7 days were 10.7 times more likely to be discharged with unfavorable management outcome than children who stayed for less than 7 days (AOR: 10.671, 95% CI: 1.363–83.546), and children who had surgical management were 2.7 times more likely than those who received conservative management to be discharged with unfavorable management outcome (AOR: 2.742, 95% CI: 1.137–6.611) (Table 3).

## 4. Discussion

This study gives an insight into management outcome and factors associated with pediatric surgical patients admitted to Arbaminch General Hospital, Southern Ethiopia. According to this study, 26% (95% confidence interval: 21%–32%) of patients were discharged with unfavorable management outcome. This result is much higher than the study done in Bangladesh 3.7% [17], South Africa 17.5%, [12], and Ethiopia [14, 16]. This disparity could be due to differences in patients' preexisting medical conditions of the patients, reasons for admission, and differences in the quality of the care.

According to the current study, male patients made up the majority of the study's admitted patients with surgical problems (59.2%). This figure was similar to those found in studies conducted in northwest Nigeria 54.9% [18] and Adama 71.9% [16]. This could be explained by the fact that male children are more prone to engage in risky behavior and that male children are more likely to have diseases like acute abdomen.

Trauma was the major cause of hospitalization in this study, which was comparable to studies conducted in Gambia [19] and Malawi [20] but differed from the studies conducted in Niger [21] and Somali land [22] that found congenital deformity to be the leading cause of admission. This disparity could be related to a disparity in hospital capacity to admit and treat or a discrepancy in sample size.

The majority of research participants (83.8%) went to the hospital after the condition had been present for more than 24 hours. This finding is in line with research conducted at Black Lion Hospital 80.4% [14]. This could be explained by the fact that the majority of patients in this study were from rural areas and were referred from other health facilities, as well as a lack of public awareness regarding health-seeking behaviors.

This study found that the mean length of hospital stay was 23.11 ± 20.826 days. This finding is relatively similar to one reported in Burundi [23]. This was much greater than a study conducted in Niger which found a mean LOS of 8.6 ± 11.2 days [21] and in Northwest Nigeria which found a mean LOS of 8.64 ± 10.31 days [18]. This could be due to differences in admission diagnoses, severity of the diseases, and quality of care.

According to the current study, surgical management was effective in 65.7% of the study participants. This result was lower than the study conducted in Black Lion Hospital (67.8%) [14] but higher than the study done in Malawi (35%) [20]. This disparity could be due to the type and severity of surgical conditions present at the time of admission, as well as hospital capacity to provide care.

The current study found that children admitted for a trauma-related reason were more likely to have an unfavorable management outcome. This is consistent with the study done in Ghana [24], Nigeria [18], and Ethiopia [25]. This could be explained by the fact that trauma cases are more likely than other admissions to have complications like infection, hemorrhage, respiratory failure, renal failure, and sepsis, which complicate surgical cases by delaying wound healing and recovery time [26].

This study found that having a preexisting medical condition at the time of admission was positively associated with unfavorable management outcome. A study conducted in Ghana and the Black Lion Hospital in Ethiopia were consistent with this finding [14, 24]. This could be explained by the fact that patients with preexisting conditions have lower immune levels, which makes them more susceptible to infection, especially nosocomial infection, which prolongs hospital stays and delays the healing of wounds. Comorbid medical conditions also increase the likelihood of inadequate food intake, which can exacerbate nutritional depletion and delay recovery from surgical conditions.

This study found that children who had an early complication at the time of admission are more likely to have unfavorable management outcome. This might be due to the fact that early complications such as bleeding reduce blood flow to vital organs, resulting in multiorgan failure and severe impairment of function organs, all of which result in unfavorable management outcome.

There has not been any previous study on this issue to compare with our findings, though.

According to this study, visiting the hospital after 24 hours of the problem's onset was associated with a poor management outcome. This result was in line with the studies done in Ethiopia [14, 25]. This could be due to the fact that late-arriving patients are more prone to complications such as sepsis, peritonitis, gangrene, and body contracture, all of which complicate surgical circumstances and management, potentially affecting the outcome.

The finding of this study showed that hospital stays of more than 7 days were significantly associated with unfavorable management outcome. This finding is supported by the studies done in China [16, 27]. This could be attributed to the fact that patients who stay in the hospital for a long time are more likely to develop bed sores, muscle weakness, rigid joints, and management-related errors, all of which affect the outcome.

According to this study, children who received surgical management were almost twice more likely to be discharged with unfavorable management outcome when compared to their counterparts. This finding was supported by a study done in Ghana [24]. This could be explained by the fact that children who have had surgery are more likely to experience complications such as postoperative infection, hemorrhage, and wound dehiscence, all of which complicate the postoperative recovery phase. They are also more likely to experience management-related errors and longer hospital stays, all of which increase the risk of complications. The results differed from those of research conducted in Malawi [20]. This disparity could be due to differences in hospital management methods, sample size, and the quality of care offered.

### 4.1. Limitation of the Study

Because the study was a retrospective, some variables, such as children's and parents' educational levels, living conditions, and parental income, were difficult to extract from the chart and may have influenced the study's outcome.

## 5. Conclusion

Trauma was the leading cause of admission in the study area, followed by surgical infection and gastrointestinal problems. The majority of patients had favorable surgical management, although roughly 26% of patients were discharged with unfavorable management outcome. Furthermore, children admitted due to trauma, children with a preexisting medical condition, children who have an early complication, going to the hospital more than 24 hours after the onset of the problem, children who stayed in the hospital for more than 7 days, and children who had surgical management are all factors that are linked to a poor outcome.

As a result, priority should be given to improving the quality of care provided to patients while also minimizing the length of stay in the hospital.

To reduce delays in hospital visits, health education should be delivered to parents or the community on a regular basis at all levels.

More prospective studies to assess pediatric surgical conditions, a type of admission, how it occurs, what are the risk factors, how severe the condition was, the quality of care provided, the socioeconomic status of parents, and the like would be preferable for researchers in order to obtain comprehensive information that is crucial for developing strategies and policies.

## Figures and Tables

**Table 1:** Sociodemographic characteristics of pediatric surgical patients admitted to Arbaminch General Hospital from January 1, 2017, to December 31, 2020 (*n* = 265).

Variables	Category	Frequency	Percent
Age	≤4	116	43.8
	5–9	56	21.1
	10–14	40	15.1
	≥15	53	20.0

Sex	Male	157	59.2
	Female	108	40.8

Residence	Urban	112	42.3
	Rural	153	57.7

**Table 2:** Causes and forms of pediatric surgical patient's admission to Arbaminch General Hospital from January 1, 2017, to December 31, 2020 (*n* = 265).

Variables	Category	Frequency	Percent
Ward of admission	Pediatric surgical	188	70.9
	Male surgical	36	13.6
	Female surgical	20	7.5
	Others^*∗*^	21	7.9

Form of admission	Elective base	91	34.3
	Emergency base	174	65.7
	Congenital anomaly	33	12.5

Admission diagnosis of children	Trauma	99	37.4
	Surgical infection	71	26.8
	Gastrointestinal problems	48	18.1
	Others^*∗*^^*∗*^	14	5.3

Gastrointestinal problems (*n* = 48)	Intussusceptions	23	47.9
	Rectal prolapsed	5	10.4
	Intestinal obstruction	11	22.9
	Pyloric stenosis	5	10.4
	Others^*∗∗∗*^	4	8.3

Diagnosed congenital anomaly (*n* = 33)	Inguinal hernia	9	27.3
	Undescended tests	7	21.1
	Club foot/lip	6	18.2
	Duodenal artesian	9	27.3
	Others^*∗∗∗∗*^	2	6.1

Diagnosed surgical infection in children (*n* = 71)	Peritonitis	13	18.3
	Appendicitis	28	39.4
	Osteomyelitis	11	15.5
	SSI	10	14.1
	Others^*∗∗∗∗∗*^	9	12.7

Others^*∗*^: pediatric medical ward, orthopedic ward, and intensive unit; Others^*∗*^^*∗*^: foreign body, bladder stone, kidney stone, submandibular cyst, and lipoma; Others^*∗∗∗*^: mesenteric lymphadenitis, enterocutaneous fistula, and pancreatic pseudocyst; Others^*∗∗∗∗*^: Hirschsprung's disease, hypospadias, midgut malrotation, and anorectal malformation; Others^*∗∗∗∗∗*^: septic arthritis, otitis media, gluteal abscess, cellulitis, and mastitis.

**Table 3:** Bivariable and multivariable logistic regression showing factors associated with the management outcome of pediatric surgical patients' admission to Arbaminch General Hospital from January 1, 2017, to December 31, 2020 (*n* = 265).

Variables	Management outcomes		COR (95% CI)	*P*-value	AOR (95% CI)	*P*-value
	Favorable	Unfavorable				
*Age*						
≤4	78 (67.2%)	38 (32.8%)	2.095 (0.951–4.615)	0.066	2.223 (0.761–6.491)	0.144
5–9	47 (83.9%)	9 (16.1%)	0.823 (0.306–2.218)	0.701	0.821 (0.239–2.824)	0.754
10–14	27 (67.5%)	13 (32.5%)	2.070 (0.797–5.378)	0.135	2.924 (0.388-10.207)	0.093
≥15	43 (81.1%)	10 (18.9%)	1	—	—	—

*Sex*						
Male	106 (67.5%)	51 (32.5%)	0.444 (0.244–0.806)	0.008	0.739 (0.326–1.676)	0.469
Female	89 (82.4%)	19 (17.6%)	1	—	—	—

*Residence*						
Rural	102 (66.7%)	51 (33.3%)	2.447 (1.347–4.446)	0.003	1.841 (0.835–4.060)	0.130
Urban	93 (83.0%)	19 (17.0%)	1	—	—	—

*Form of admission*						
Emergency base	122 (70.1%)	52 (29.9%)	0.579 (0.315–1.064)	0.078	0.840 (0.358–1.970)	0.688
Elective base	73 (80.2%)	18 (19.8%)	1	—	—	—

*Admission diagnosis*						
Trauma cause	54 (54.5%)	45 (45.5%)	4.700 (2.629–8.402)	0.000	5.753 (2.366–13.987)^*∗*^	0.000
Nontrauma	141 (84.9%)	25 (15.1%)	1	—	—	—

*Any preexisting medical condition*						
Yes	32 (47.8%)	35 (52.2%)	5.094 (2.788–9.306)	0.000	3.240 (1.436–7.310)^*∗*^	0.005

No	163 (82.3%)	35 (17.7%)	1	—	—	—

*Nutritional status*						
Well-nourished	167 (81.9%)	37 (18.1%)	1	—	—	—
Mall-nourished	28 (45.9%)	33 (54.1%)	0.188 (0.101–0.348)	0.000	0.555 (0.237–1.296)	0.174

*Any early complication*						
Yes	65 (56.5%)	50 (43.5%)	5.094 (2.788–9.306)	0.000	2.515 (1.130–5.599)^*∗*^	0.024
No	130 (86.7%)	20 (13.3%)	1	—	—	—

*Duration of time before the presentation*						
<24 hr	39 (90.7%)	4 (9.3%)	1	—	—	—
≥24 hr	156 (70.3%)	66 (29.7%)	4.125 (1.417–12.008)	0.009	8.351 (2.089–33.381)^*∗*^	0.003

*LOS (length of hospital stays)*						
<7 days	52 (98.1%)	1 (1.9%)	1	—	—	—
≥7 days	143 (67.5%)	69 (32.5%)	25.091 (3.397–185.299)	0.002	10.671 (1.363–83.546)^*∗*^	0.024

*Hospital management*						
Surgical	120 (69.0%)	54 (31.0%)	2.109 (1.126–3.953)	0.020	2.742 (1.137–6.611)^*∗*^	0.025
Conservative	75 (82.4%)	16 (17.6%)	1	—	—	—

*Note*. COR = crude odds ratio, AOR = adjusted odds ratio, CI = confidence interval, LOS = length of hospital stay, and ^*∗*^*P*-value <0.05.

## Data Availability

The datasets used and/or analyzed during the current study are available from the corresponding author upon reasonable request.

## Abbreviations

AAU: Addis Ababa University
AIDS: Acquired immune deficiency syndrome
AOR: Adjusted odds ratio
ART: Antiretroviral therapy
CI: Confidence interval
COR: Crude odds ratio
DM: Diabetes mellitus
LOS: Length of hospital stay
LMIC: Low- and middle-income countries
OPD: Outpatient department
PI: Principal investigator
SSI: Surgical site infection
SNNPR: South Nation Nationality and People Region
SPSS: Statistical package of social science
UN-IGME: United Nations Interagency Group for Child Mortality Estimation
WHO: World Health Organization.

## Additional Points

*Authors Information*. Filagot Assefa: MSc in Pediatrics and Child Health Nursing, Arbamich Health Science College, Arbaminch. Fikre Moga: MSc in Pediatrics and Child Health Nursing, School of Nursing, College of Medicine and Health Science, Arba Minch University. Kalkidan Wondwossen: MSc in Pediatrics and Child Health Nursing, Assistant Professor; School of Nursing and Midwifery, College of Health Science, Addis Ababa University. Yeshiwork Berhan: MSc, School of Nursing & Midwifery, College of Health Science, Addis Ababa University.
